# Global Scientific Outputs of Microsurgery Publications: A Bibliometric Approach About Yesterday, Today, and Tomorrow

**DOI:** 10.7759/cureus.12205

**Published:** 2020-12-21

**Authors:** Serkan Guler, Ramadan Ozmanevra, Sercan Çapkin

**Affiliations:** 1 Orthopaedics and Traumatology, Aksaray University, Faculty of Medicine, Aksaray, TUR; 2 Orthopaedics and Traumatology, University of Kyrenia, Dr. Suat Gunsel Hospital, Kyrenia, CYP

**Keywords:** bibliometric analysis, microsurgery, citation analysis, web of science

## Abstract

Introduction

Although there have been important developments in microsurgery in recent years, there is no current and comprehensive bibliometric study in the literature. In this study, we aimed to present a summary of the articles published on microsurgery between 1980 and 2019 with bibliometric analysis.

Methods

Articles published on microsurgery between 1980 and 2019 were withdrawn from the Web of Science database and analyzed by bibliometric methods. Citation analysis was performed to identify effective journals and articles. Keyword cluster and trends analyses were performed for a detailed analysis of the researched topics. Relationships between the article numbers of the countries and gross domestic product (GDP) and human development index (HDI) values were investigated using Spearman’s correlation coefficient. A linear regression analysis was used to estimate the number of articles to be published in the future.

Results

A total of 3,537 publications related to microsurgery were found. Bibliometric analyses were performed in 2,063 articles (58.3%) of these publications. The most active countries in publishing were the United States (504), Germany (286), and Italy (154), respectively. A statistically significant correlation was found between the article numbers and the GDP and HDI sizes of the countries (r = 0.758, p <0.001, r = 0.659, p <0.001).

Conclusion

The economic size and development levels of the countries were an important factor in academic productivity in microsurgery. Undeveloped countries should be encouraged by performing multidisciplinary studies in this regard.

## Introduction

Microsurgery is a term used to describe the surgical techniques that require surgical microscopy and the necessary special instrumentation (microscopes, microstrums, and micro-sutures) to perform sensitive operations on small structures in our body [1]. It specializes in many clinical disciplines including microsurgical orthopedics, otorhinolaryngology, ophthalmology, neurosurgery, plastic surgery, transplantation surgery, oncology, gynecology, and urology [2]. There are highly efficient microsurgery applications in orthopedic surgery [3].

A bibliometric analysis involves analyzing scientific publications, such as published articles, books, congress abstracts, etc., using statistical methods. Although the number of scientific publications is increasing every day, access to medical literature is becoming easier. Therefore, it is difficult to search the literature for researchers who want to perform a new study on a subject. There is an increase in bibliometric studies in the field of health, in parallel with the significant increase in the number of publications in recent years. Researchers can easily access the summary information of thousands, sometimes tens of thousands, of articles published in a research area on a particular subject, through bibliometric studies. In addition, the most influential articles, active universities, authors, countries, and international collaborations can be determined in a short time. Important bibliometric studies have been conducted in many different disciplines in the field of health. Recently, some studies have been conducted in the field of anesthesia, neurosurgery, rhinoplasty, and general surgery [4-10].

Although there have been important developments in microsurgery in recent years, there is no current and comprehensive bibliometric study in the literature.

In this study, we aimed to present a summary of the articles published on microsurgery between 1980 and 2019 with bibliometric analysis. In addition, we aimed to reveal the most cited effective publications, active journals, countries, institutions, and authors; identify international collaborations; and discuss keyword analysis and trending research topics. We also aimed to determine the development of publications over the years and the factors affecting the productivity of the publication with correlation and regression analysis.

## Materials and methods

The literature review was performed using the Web of Science (WoS; Clarivate Analytics, Philadelphia, Pennsylvania) database (access date: May 10, 2020). All articles published on microsurgery were searched. All the publications with the keywords “microsurgery/micro surgery/micro-surgery” in the title and published between 1980 and 2019 were pulled from the WoS database and analyzed by bibliometric methods. The codes for repeatability were as follows: Title: (“micro-surgery”) OR Title: (“micro surgery”) OR Title: (microsurgery) and Document Types: (Article) Timespan = 1980-2019. Indexes = SCI-Expanded, SSCI, A & HCI, CPCI-S, CPCI-SSH, BKCI-S, BKCI-SSH, ESCI). Bibliometric network visualizations were performed using the VOSviewer (Version 1.6.15; Centre for Science and Technology Studies, The Netherlands) package program [11].

Statistical analyses were performed using the Statistical Package for the Social Sciences (SPSS) (Version 22.0, IBM Corp., Armonk, NY) package programming. The normality of data distribution was tested using the Shapiro-Wilk test. The correlation between the number of articles produced by countries and gross domestic product (GDP) (data obtained from the World Bank Group website) [12] and human development index (HDI) (data from the United Nations Development Program Human Development Report 2019) [13] was determined using Spearman’s correlation coefficient in accordance with the data distribution. Linear regression analysis was used to estimate the number of articles to be published in the future. A p-value of <0.05 was considered significant.

## Results

As a result of the literature review, 3,537 publications were found. A total of 2,063 (58.32%) of these publications were articles, 441 (12.46%) were proceedings papers, 430 (12.15%) were meeting abstracts, 315 (8.90%) were editorial materials, 186 (5.25%) were letters, and 151 (4.26%) were reviews. The rest were other types of publications: note (46), book chapter (31), correction (12), discussion (8), news item (8), book review (7), biographical item (4), reprint (4), book (3), and correction addition (1). In our study, 2,063 articles were analyzed bibliometrically in the article category. A total of 1,759 (85.2%) of the articles were published in English, and the rest were published in other languages (German: 139, French: 95, Russian: 37, Spanish: 14, Japanese: 6, Italian: 3, Korean: 3, Czech: 2, Portuguese: 2, Dutch: 1, Greek: 1, and Turkish: 1). A total of 2,063 articles had 37,577 citations (without self-citations: 33,493), the average number of citations was 18.21, and the h-index was 86.

Research areas

More than half of the published articles related to microsurgery were in the field of surgery (1075; 52.1%). Most of the articles published were in the areas of otorhinolaryngology (254), clinical neurology (221), gastroenterology hepatology (147), medicine general internal (87), obstetrics-gynecology (84), ophthalmology (74), dentistry oral surgery medicine (69), medicine research experimental (67), oncology (59), radiology nuclear medicine medical imaging (57), neurosciences (43), urology nephrology (43), orthopedics (42), and optics (35). Some of the articles were labeled in more than one field.

Development of publications

**Figure 1:**
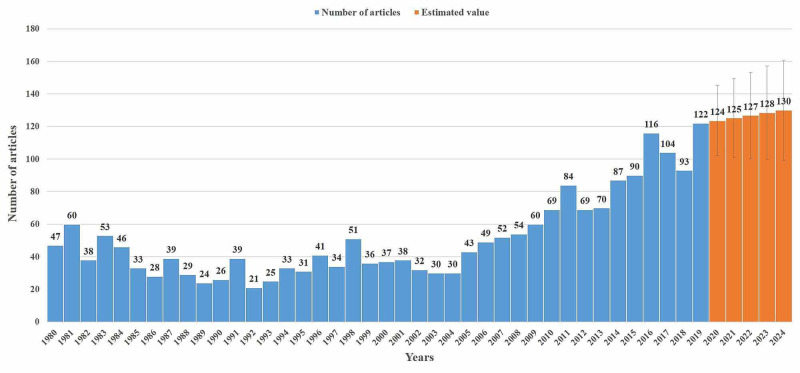
Distribution of the number of articles on microsurgery by year Footnote: The orange color shows an estimate of the number of articles to be published in the coming years.

The distribution of articles by year is presented in Figure 1. The number of publications for the next five years predicted by regression analysis is also shown in Figure 1 with 95% confidence intervals. The estimated number of publications was 124 (102-145) for 2020 and 130 (99-161) for 2024.

Trend topics

**Figure 2:**
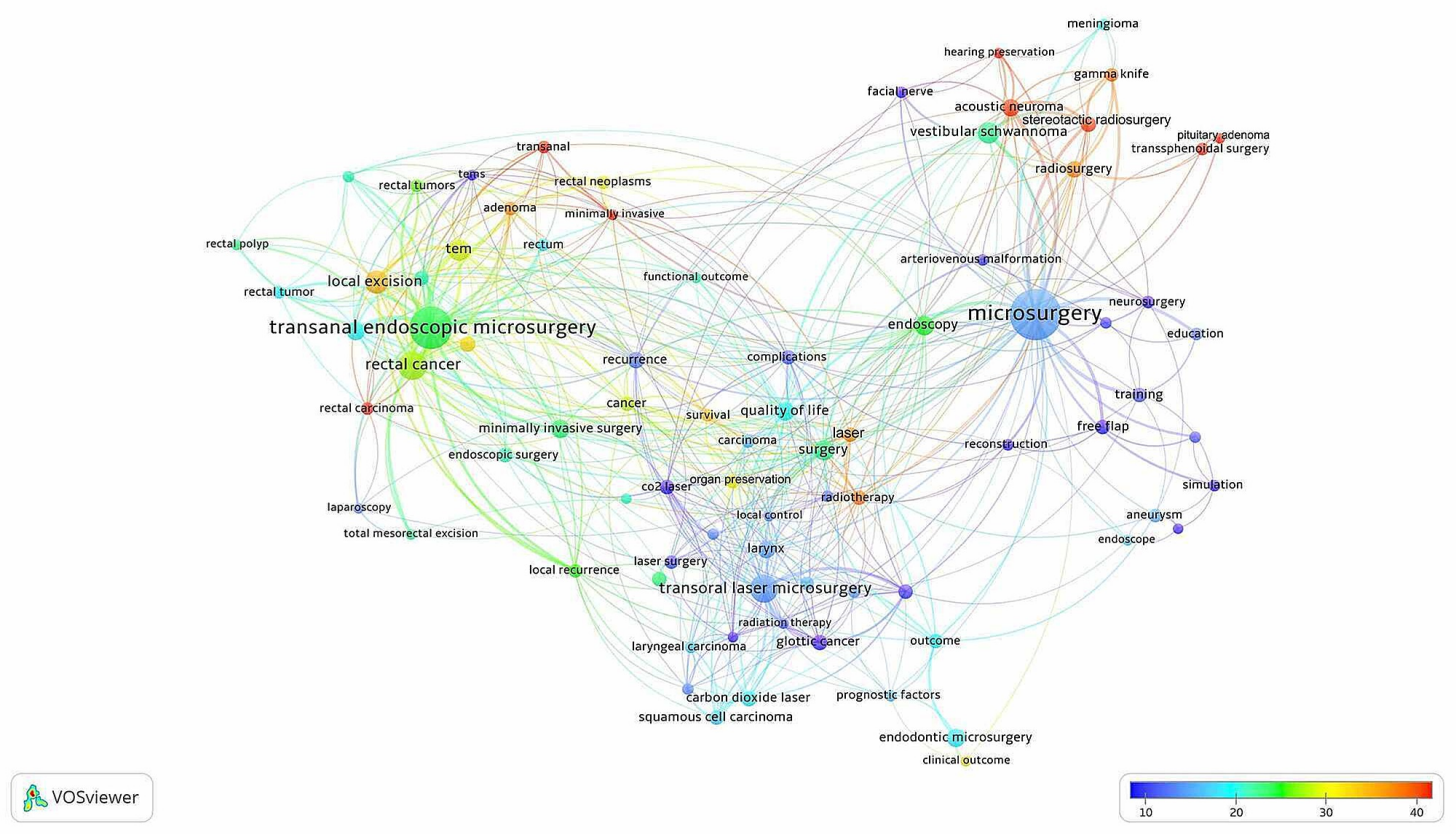
Network visualization map for citation analysis of keywords on microsurgery Footnote: The number of citations taken by keyword increases from blue to red (blue-green-yellow-red). The size of the circles indicates that the keyword is used frequently. The thickness of the lines indicates the strength of the relationship.

A total of 2,628 different keywords were used in 2,063 articles. Approximately 76 keywords used in at least eight different articles from these keywords are listed in Table 1. The network visualization map obtained according to the citation analysis of the articles containing the keywords is shown in Figure 2.

**Table 1:** The first 76 trend keywords on microsurgery The first 76 trend keywords on microsurgery

Keyword	O	Keyword	O	Keyword	O	Keyword	O
microsurgery	311	laser microsurgery	19	aneurysm	13	glottic carcinoma	9
transanal endoscopic microsurgery	208	glottic cancer	18	carcinoma	13	glottis	9
rectal cancer	82	stereotactic radiosurgery	18	early glottic cancer	13	head and neck cancer	9
transoral laser microsurgery	74	cancer	17	organ preservation	13	meningioma	9
local excision	47	free flap	17	rectal neoplasms	13	reconstruction	9
tem(s)	47	squamous cell carcinoma	17	education	12	simulation	9
vestibular schwannoma	39	training	17	neurosurgery	12	clinical outcome	8
surgery	34	co2 laser	16	rectum	12	clipping	8
endoscopy	30	laryngeal cancer	16	transanal	12	endoscope	8
quality of life	29	outcome	16	transsphenoidal surgery	12	functional outcome	8
rectal adenoma	27	complications	15	rectal carcinoma	11	hearing preservation	8
endodontic microsurgery	26	early rectal cancer	15	survival	11	laparoscopy	8
minimally invasive surgery	26	endoscopic surgery	15	anastomosis	10	local control	8
larynx	25	laser	15	arteriovenous malformation	10	minimally invasive	8
acoustic neuroma	24	radiotherapy	15	facial nerve	10	pituitary adenoma	8
rectal tumor(s)	23	adenoma	14	laryngeal carcinoma	10	radiation therapy	8
radiosurgery	22	gamma knife	14	prognostic factors	10	rectal polyp	8
recurrence	20	laser surgery	14	reconstructive surgery	10	total mesorectal excision	8
carbon dioxide laser	19	local recurrence	14	transanal excision	10	voice	8

Active authors

Authors who published the most articles on microsurgery were Steiner W (28), De Graaf EJR (24), Campisi C (20), Boccardo F (18), Morino M (17), Arezzo A (15), Allaix ME (14), Doornebosch PG (14), Hinni ML (14), and Lezoche E (14).

Active organization

The 16 organizations and organizations-enhanced that published the most articles on microsurgery are presented in Table 2.

**Table 2:** Active organization and organizations-enhanced on microsurgery

Organizations	RC	Organizations-Enhanced	RC
University Gottingen	34	Assistance Publique Hopitaux Paris (APHP)	42
University Genoa	30	University of California System	39
Mayo Clinic	24	University of Gottingen	39
Ijsselland Hospital	22	Harvard University	35
Harvard University	21	Mayo Clinic	33
Johns Hopkins University	21	University of Genoa	33
University Penn	21	University of Texas System	27
Leiden University	17	Johns Hopkins University	24
Stanford University	17	University of Pennsylvania	23
University Calif Irvine	17	Ijsselland Hospital	22
University Roma La Sapienza	16	Sapienza University Rome	22
University Turin	16	University of Munich	22
Yonsei University	16	University of London	21
Louisiana State University	14	Erasmus University Rotterdam	20
University Milan	14	Pennsylvania Commonwealth System of Higher Education (PCSHE)	20
University Toronto	14	University of Erlangen Nuremberg	19

Active journals

A total of 636 journals with 2,063 articles were published. Among these journals, 74 published at least six articles on this subject. The first 74 journals that produced the most publications are presented in Table 3. The table lists the total number of citations and the average number of citations per article.

**Table 3:** Active journals on microsurgery

Journals	RC	C	AC	Journals	RC	C	AC
Journal of Reconstructive Microsurgery	84	330	3.9	Frontiers in Oncology	10	30	3.0
Microsurgery	78	1392	17.8	Helvetica Chirurgica Acta	10	2	0.2
Surgical Endoscopy and Other Interventional Techniques	57	2107	37.0	Otology & Neurotology	9	73	8.1
Plastic and Reconstructive Surgery	43	1281	29.8	Klinische Monatsblatter Fur Augenheilkunde	9	29	3.2
Neurosurgery	35	1884	53.8	International Surgery	9	26	2.9
Laryngoscope	34	965	28.4	American Journal of Surgery	8	365	45.6
Journal of Endodontics	34	880	25.9	Journal of Otolaryngology-Head & Neck Surgery	8	107	13.4
Colorectal Disease	34	802	23.6	Journal of Hand Surgery-American Volume	8	105	13.1
Head and Neck-Journal for the Sciences and Specialties of the Head and Neck	34	799	23.5	Clinical Otolaryngology	8	93	11.6
European Archives of Oto-Rhino-Laryngology	30	423	14.1	Surgical Laparoscopy Endoscopy & Percutaneous Techniques	8	66	8.3
Annals of Plastic Surgery	30	273	9.1	International Journal of Medical Robotics and Computer Assisted Surgery	8	55	6.9
Acta Neurochirurgica	27	776	28.7	Acta Oto-Laryngologica	8	49	6.1
Journal of Neurosurgery	26	1909	73.4	Therapeutische Umschau	8	2	0.3
Diseases of The Colon & Rectum	25	1682	67.3	Surgical Neurology	7	417	59.6
Handchirurgie Mikrochirurgie Plastische Chirurgie	19	78	4.1	Surgical Endoscopy-Ultrasound and Interventional Techniques	7	319	45.6
International Journal of Colorectal Disease	16	413	25.8	Journal of Gastrointestinal Surgery	7	155	22.1
Otolaryngology-Head and Neck Surgery	16	346	21.6	Journal of Oral and Maxillofacial Surgery	7	150	21.4
Clinics in Plastic Surgery	16	159	9.9	International Journal of Computer Assisted Radiology and Surgery	7	110	15.7
HNO	16	97	6.1	Journal of Biomedical Optics	7	91	13.0
Vestnik Oftalmologii	15	12	0.8	Lasers in Surgery and Medicine	7	84	12.0
British Journal of Surgery	14	854	61.0	Chirurg	7	79	11.3
World Neurosurgery	14	55	3.9	Lymphology	7	62	8.9
Archives of Gynecology and Obstetrics	14	38	2.7	British Journal of Plastic Surgery	7	42	6.0
Fertility and Sterility	13	457	35.2	Annales De Chirurgie Plastique Esthetique	7	16	2.3
Techniques in Coloproctology	13	91	7.0	Khirurgiya	7	1	0.1
Minimally Invasive Therapy & Allied Technologies	13	89	6.8	Vestnik Khirurgii İmeni II Grekova	7	0	0.0
Annals of Otology Rhinology and Laryngology	12	299	24.9	World Journal of Surgery	6	161	26.8
Journal of Laparoendoscopic & Advanced Surgical Techniques	12	56	4.7	Acta Orthopaedica Scandinavica	6	115	19.2
Plastic and Reconstructive Surgery-Global Open	12	23	1.9	Neurosurgical Focus	6	90	15.0
Neurochirurgie	11	86	7.8	Hepato-Gastroenterology	6	65	10.8
Laryngo-Rhino-Otologie	11	51	4.6	Chinese Medical Journal	6	51	8.5
Journal Francais D Ophtalmologie	11	19	1.7	Plos One	6	45	7.5
Biomedical Optics Express	10	219	21.9	Medicine	6	16	2.7
Journal of Voice	10	177	17.7	Neurological Surgery	6	13	2.2
Journal of Plastic Reconstructive and Aesthetic Surgery	10	157	15.7	Acta Chirurgica Belgica	6	9	1.5
Minimally Invasive Neurosurgery	10	121	12.1	Australian and New Zealand Journal of Surgery	6	4	0.7
Journal of Laryngology and Otology	10	49	4.9	Sciences Et Techniques De L Animal De Laboratoire	6	0	0.0

Active countries

**Figure 3:**
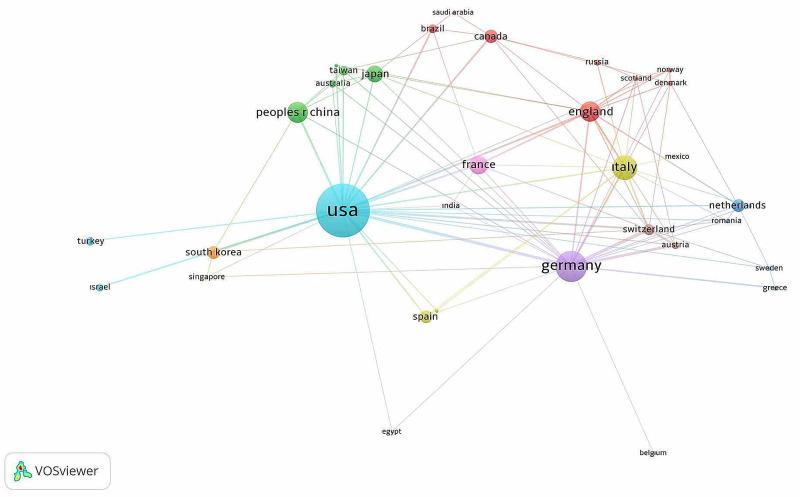
Network visualization map for the international collaboration of worldwide countries on microsurgery Footnote: The size of the circle shows the large number of publications, the colors indicate the cluster of collaboration, and the thickness of the lines indicates the strength of the collaboration.

A total of 2,063 articles were published, with a total of 85 country addresses. Active countries producing more than 20 publications are the United States (US) (504), Germany (286), Germany (52), Italy (154), United Kingdom (UK) (145), France (139), China (113), Japan (81), Switzerland (69), Canada (65), Spain (60), Netherlands (58), South Korea (52), Belgium (43), Australia (41), Austria (37), Taiwan (34), Brazil (31), Turkey (28), Israel (24), Russia (21), and Union of Soviet Socialist Republics (37). The network map showing at least five publications from these countries and showing international cooperation among the 34 countries are illustrated in Figure 3.

Correlation analysis

**Figure 4:**
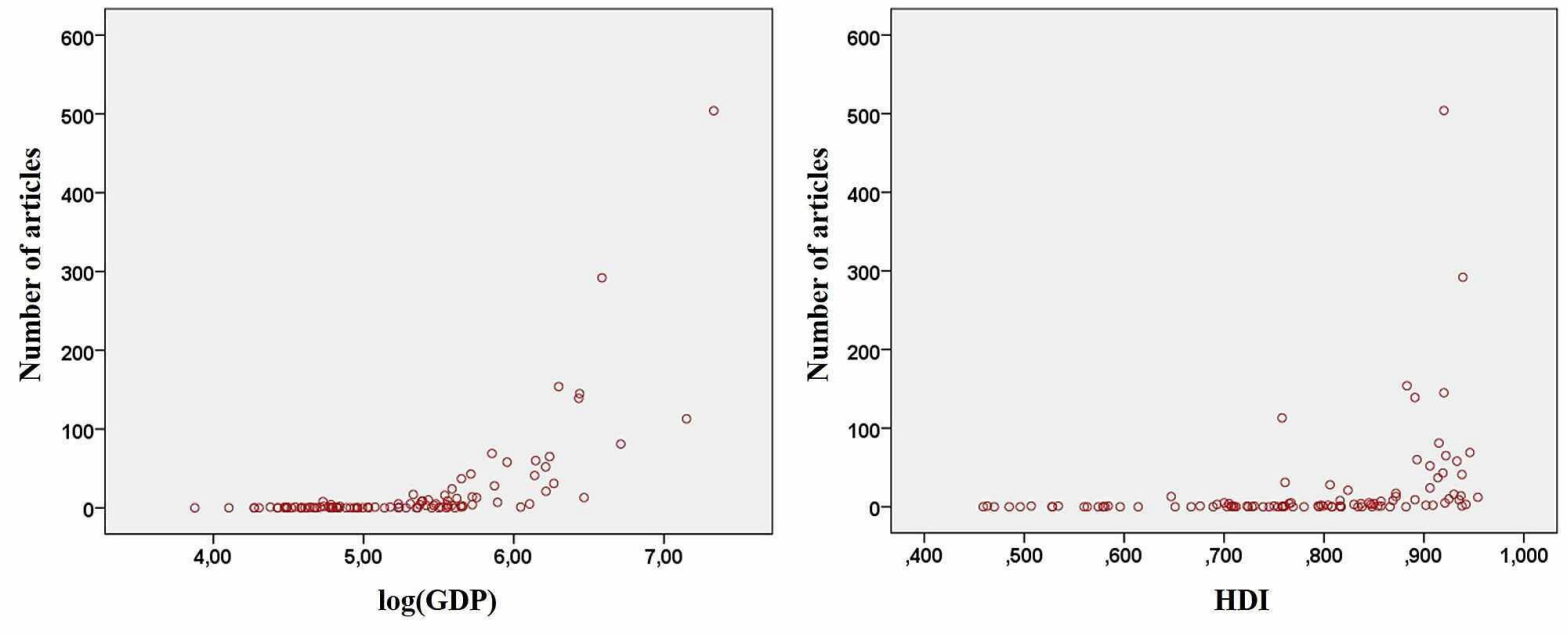
Scatter plot between the number of articles produced by country on microsurgery and log (GDP) and HDI values Footnote: GDP: gross domestic product, HDI: human development index

A statistically significant correlation was found between the number of articles published by countries on microsurgery and the GDP and HDI sizes of countries (r = 0.758, p <0.001, r = 0.659, p <0.001). Scatter plots of correlations are presented in Figure 4.

Co-citation analysis

In the references section of 2,063 articles, 29,387 publications were referred. Among these were eight publications that received more than 50 citations. These most cited publications were Buess (1984) (number of citations: C; 114), Winde (1996) (C: 86), Middleton (2005) (C: 68), Heintz (1998) (C: 60), Moore (2008) (C: 56), Lee (2003) (C: 53), Mellgren (2000) (C: 52), and Buess (1992) (C: 51) [14-21].

Citation analysis

When the analyzed articles were evaluated according to the total number of citations and the average number of citations per year, the most cited study was Anderson et al.’s study titled “Selective photothermolysis - precise microsurgery by selective absorption of pulsed radiation” published in Science in 1983 [22]. The remaining most cited articles were performed by Sylla et al. [23], Haughey et al. [24], Hutson et al. [25], and Bach et al. [26], respectively. The articles were ranked according to the average number of citations per year, and the first five articles are presented in Table 4 [22-26]. The total citation numbers of the articles are also provided in the table.

**Table 4:** The five most cited manuscripts on microsurgery

No	Article	Author	Journal	PY	TC	AC
1	Selective photothermolysis - precise microsurgery by selective absorption of pulsed radiation	Anderson, RR. et al. [22]	Science	1983	1952	51.37
2	Notes transanal rectal cancer resection using transanal endoscopic microsurgery and laparoscopic assistance	Sylla, P. et al. [23]	Surgical Endoscopy and Other Interventional Techniques	2010	312	28.36
3	Transoral laser microsurgery as primary treatment for advanced-stage oropharyngeal cancer: a united states multicenter study	Haughey, Bruce H. et al. [24]	Head and Neck-Journal for the Sciences and Specialties of the Head and Neck	2011	209	20.9
4	Forces for morphogenesis investigated with laser microsurgery and quantitative modeling	Hutson, MS. et al. [25]	Science	2003	344	19.11
5	A predictive model for local recurrence after transanal endoscopic microsurgery for rectal cancer	Bach, SP. et al. [26]	British Journal of Surgery	2009	213	17.75

## Discussion

According to our comprehensive statistical analysis findings with 2,063 articles, a significant increase was observed in the number of articles on microsurgery especially after 2005. The number of articles published ranged from 21 to 60 per year between 1980 and 2009. The number of articles exceeded 60 in 2010 and reached 122 in 2019. When the regression analysis results were evaluated, we observed that the number of articles published will continue to increase.

When the results of the keyword analysis were evaluated, the top 10 topics related to microsurgery investigated were transanal endoscopic microsurgery (TEM), rectal cancer/adenoma, transoral laser microsurgery, local excision, vestibular schwannoma, endoscopy, quality of life, endodontic microsurgery, minimally invasive surgery, and larynx. According to the results of cluster analysis, four major primary clusters were formed based on the topics used in similar articles: microsurgery, transanal endoscopic microsurgery/rectal cancer/local excision, transoral laser microsurgery/quality of life, and vestibular schwannoma/acoustic neuroma. The literature on microsurgery developed four main clusters, with a total of eight different clusters.

According to the results of trend analysis to determine the current research topics, issues related to transanal endoscopic microsurgery, rectal carcinoma/tumors, laser microsurgery, endoscopy, transsphenoidal surgery, gamma knife, and pituitary adenoma were initially studied; in the following years, acoustic neuroma, stereotactic radiosurgery, radiotherapy, arteriovenous malformation, local excision, minimally invasive, transanal, rectal neoplasms, rectal polyp, laparoscopy, facial nerve, hearing preservation, neurosurgery, anastomosis, vestibular schwannoma, reconstructive surgery, meningioma, aneurysm, clipping, larynx, survival, carcinoma, local recur, carcinoma, carbon dioxide laser, squamous cell carcinoma, early glottic cancer, and organ preservation were studied. In recent years, subjects such as transoral laser microsurgery, quality of life, training, education, free flap, simulation, endodontic microsurgery, recurrence, CO2 laser, head and neck cancer, outcome, glottis cancer, voice, glottis, and local control have been explored. The keywords used in more cited articles were acoustic neuroma, stereotactic radiosurgery, hearing preservation, transsphenoidal surgery, pituitary adenoma, minimally invasive, transanal, rectal carcinoma, gamma knife, radiosurgery, radiotherapy, laser, adenoma, and local excision.

The most active journals producing more than 30 publications were the Journal of Reconstructive Microsurgery, Microsurgery, Surgical Endoscopy and Other Interventional Techniques, Plastic and Reconstructive Surgery, Neurosurgery, Laryngoscope, Journal of Endodontics, Colorectal Disease, and Head and Neck-Journal for the Sciences and Specialties of the Head and Neck. Researchers who want to publish manuscripts on this subject can consider these journals. When the journals were evaluated according to the number of citations per article, Journal of Neurosurgery, Diseases of the Colon & Rectum, British Journal of Surgery, Surgical Neurology, Neurosurgery, American Journal of Surgery, Surgical Endoscopy-Ultrasound and Interventional Techniques, Surgical Endoscopy, and Other Interventional Techniques journals came to the fore as the more cited journals. Researchers who want their articles to be cited more can first consider these journals.

When the publication distributions of the world countries were analyzed, we found that developed countries produce the most publications in microsurgery (US, Germany, Italy, UK, France, Japan, Switzerland, Canada, Spain, Netherlands, South Korea, Belgium, Australia, Austria, Taiwan, and Israel) or developing countries with large economies (China, Brazil, Turkey, and Russia). When the relation graph plotted for the correlations was evaluated, countries with HDI 0.88 and above provided remarkable article contributions. Countries with a log (GDP) value of more than 5.6 (about 400,000 GDP) contributed significantly to the attention. Some studies conducted in the literature reported that the economic size or development levels of the countries had a significant effect on academic publication productivity [4-5]. Some cutting points were discussed in our study. When co-authoring cooperation of countries was evaluated, we found that geographical regional neighborhood is an important factor in cooperation.

When the analyzed articles were evaluated according to the total number of citations and the average number of citations per year, the most cited study was Anderson et al.’s study titled “Selective photothermolysis - precise microsurgery by selective absorption of pulsed radiation” published in Science in 1983 [22]. The remaining most cited articles were presented in Table 4 [23-26]. Buess (1984), Winde (1996), Middleton (2005), Heintz (1998), Moore (2008), Lee (2003), Mellgren (2000), and Buess (1992) were cited in all the articles analyzed according to the co-citation analysis findings [14-21]. Researchers interested in this subject should read these studies, which are determined primarily by attribution and co-citation analysis.

In our study, comprehensive bibliometric analyses, such as keyword analysis, citation analysis for articles and journals, international collaborations, and correlation analyses, were performed for the first time in this study. Our study is the most comprehensive research in the literature in which the most articles are analyzed on this subject.

This study has some limitations. It only reviewed the articles published in the WoS database. The PubMed and Scopus databases were not included for analysis. In the bibliometric studies where many articles were analyzed, if multiple databases are used, the same articles (there maybe thousands) in different databases can be included in the analysis twice. This will negatively affect the reliability of the results.

## Conclusions

As a result of our study on microsurgery, which has an increase in the number of articles every day in the literature, summary information of 2,063 articles published between 1980 and 2019 was presented. The most active publishing countries were the US, Germany, and Italy. There was a significant correlation between article numbers and countries’ GDP and HDI sizes. The top three journals to publish articles were the Journal of Reconstructive Microsurgery, Microsurgery, and Surgical Endoscopy and Other Interventional Techniques. The most active institution was University Gottingen.

The researchers will be able to get ideas for new studies on this subject by evaluating the development of the topics studied by year, the trending topics, and the topics that received more citations. Hence, this study will be a useful guide for clinicians and scientists on the global outcomes of studies on microsurgery.
